# Learning Statistical Models for Annotating Proteins with Function Information using Biomedical Text

**DOI:** 10.1186/1471-2105-6-S1-S18

**Published:** 2005-05-24

**Authors:** Soumya Ray, Mark Craven

**Affiliations:** 1 Department of Computer Sciences, University of Wisconsin, Madison, Wisconsin 53706, USA; 2 Department of Biostatistics and Medical Informatics, University of Wisconsin, Madison, Wisconsin 53706, USA

## Abstract

**Background:**

The BioCreative text mining evaluation investigated the application of text mining methods to the task of automatically extracting information from text in biomedical research articles. We participated in Task 2 of the evaluation. For this task, we built a system to automatically annotate a given protein with codes from the Gene Ontology (GO) using the text of an article from the biomedical literature as evidence.

**Methods:**

Our system relies on simple statistical analyses of the full text article provided. We learn *n*-gram models for each GO code using statistical methods and use these models to hypothesize annotations. We also learn a set of Naïve Bayes models that identify textual clues of possible connections between the given protein and a hypothesized annotation. These models are used to filter and rank the predictions of the *n*-gram models.

**Results:**

We report experiments evaluating the utility of various components of our system on a set of data held out during development, and experiments evaluating the utility of external data sources that we used to learn our models. Finally, we report our evaluation results from the BioCreative organizers.

**Conclusion:**

We observe that, on the test data, our system performs quite well relative to the other systems submitted to the evaluation. From other experiments on the held-out data, we observe that (i) the Naïve Bayes models were effective in filtering and ranking the initially hypothesized annotations, and (ii) our learned models were significantly more accurate when external data sources were used during learning.

## Introduction

We participated in the first two subtasks of Task 2 of the BioCreative text mining evaluation. The overall task was designed to evaluate methods for automatically annotating proteins with codes from the Gene Ontology (GO) [1] using articles from the scientific literature. In the first subtask (2.1), a system is given a document, an associated protein and a GO code, and is asked to return a segment of text from the document that supports the annotation of the text with the GO code (the *evidence text*). In the second subtask (2.2), a system is given a document and an associated protein, and is asked to return all GO codes that the pair should be annotated with, along with the associated evidence text for each GO code.

Our approach to the annotation task is based on a statistical machine learning perspective. It is fairly straightforward and incorporates little in the way of linguistic and biological knowledge. It does, however, leverage several existing on-line biological resources, including the MeSH dictionary of biological terms, and databases providing protein-name aliases and GO annotations for proteins. We believe that our approach serves as a useful "baseline" approach, whose performance in the annotation task can likely be improved by the addition of expert biological and linguistic knowledge.

Several key issues need to be addressed to effectively solve Task 2. First, it is unlikely that the exact strings of many GO codes occur in the text that is used to annotate query proteins. It is more likely that the relevance of particular GO codes must be inferred from indirect descriptions that we see in the text. Therefore, for most GO codes, we learn models that infer their relevance by looking for related terms. Learning these models, however, calls for more text associated with each GO code than what is available in the Task 2 training set. To address this issue, we collect data from several publicly available databases that describe GO annotations of protein-document pairs for other non-human organisms, such as yeast and Drosophila. A second issue is that even when our GO code models suggest that a GO code might be inferred from a passage of text, we need to evaluate whether this GO code is related to the protein of interest. To do this, we learn statistical models to discriminate between passages of text that relate proteins to GO codes from those that do not. A third key issue is that the relevant passages of text are not marked in the training set. In our approach, we simply assume that all passages that mention the protein and a GO code in a training document do in fact relate the protein to the GO code.

## System description

Processing document-protein queries in our system involves several key steps:

• The documents are pre-processed into a standardized representation.

• The documents are then scanned for occurrences of query proteins. This step involves the use of a protein-alias database and a set of heuristic rules for protein-name matching.

• Selected passages of the documents are then scanned for matches against GO codes. This step employs statistical models of GO codes that are learned from training-set documents.

• Text passages containing putative matches against the query protein and against GO codes are filtered and ranked by a learned statistical model. These models are trained to discriminate between passages that relate GO codes to proteins and passages that do not.

**Figure 1:**
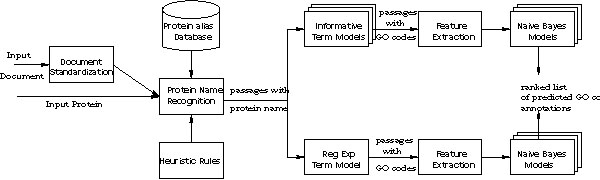
**System block diagram for Task 2.2. **There is one Informative Term model for every GO code with sufficient training data. There are six Naïve Bayes models: one for each ontology and for each method of GO code prediction.

In the following sections, we describe each step in detail. Figure 1 shows a block diagram representation of the overall system.

### Standardizing documents

**Figure 2:**
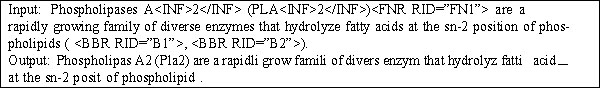
**Example of document standardization. **The input line is a sentence from an XML-formatted Journal of Biological Chemistry (*JBC*) document. The output line shows the result after standardization.

The first step performed by our system is to transform a given document into a standardized token-based representation. We first strip all XML tags from the document, while retaining the paragraph structure. We also remove all text outside the abstract and main body of the document. All HTML ampersand codes are transformed to their ASCII equivalents. Next, we remove extraneous whitespace and stem all words using the Porter stemmer [2]. We then transform species names into a common expanded format using a hand-built dictionary of such names. We also split common hyphenated compound words into their constituents using a dictionary of suffixes. Finally, we use a dictionary of biomedical terms from MeSH [3] to represent technical compound terms when they occur in a given document. Figure 2 shows an example of the input text before and after standardization.

### Recognizing protein names

In order to annotate document-protein pairs with GO codes, we must first find references to a given protein in the document. We do this by searching for the given protein name as well as aliases gathered from SwissPROT [4] and HuGO [5]. When matching an alias (including the given name) to a piece of text, we use a simple regular-expression representation of the alias as well as the literal string. These regular expressions allow for variations in punctuation and special characters in the matched text.

If we do not find any matches to the given protein name or its aliases, then we search using a set of "approximate aliases" that are generated by applying a set of simple heuristics to the given aliases. Some examples of these heuristics are rules that strip off trailing words (e.g., *protein *and *fragment*), and rules that attempt to reduce a specific protein name to a family name (e.g., by dropping a one-character token at the end of a given name).

### Recognizing GO codes

In addition to finding references to proteins, our system must also find references to Gene Ontology codes. In many cases, however, we expect that relevant GO code names will not appear verbatim in the articles being processed. Therefore, we construct statistical models to predict whether each GO code is associated with a protein-document pair. In particular, we learn a model for each GO code for which we have sufficient training documents. Since the provided training set is very small and represents relatively few GO codes, we use databases from the GO Consortium website to gather more data. The databases we use include SGD (yeast) [6], FlyBase (Drosophila) [7], WormBase (C. elegans) [8] and TAIR (Arabidopsis) [9]. They are similar to the GOA database [10] given to us in that they list protein, GO code and document identifier triplets for many proteins belonging to the respective organisms. We extract those triplets from these databases in cases where the associated documents have PubMed identifiers attached to them. Then, we obtain the abstracts for these documents from MEDLINE. We consider this text to be "weakly labeled" with GO codes because it is possible that the evidence associating a GO code of interest to the protein might not be mentioned in the abstract. However, we hypothesize that if we collect significant numbers of documents for any GO code, a large enough fraction will contain this type of evidence, thereby allowing us to learn an accurate model for that GO code. We refer to our models for GO codes as Informative Term models.

**Figure 3:**
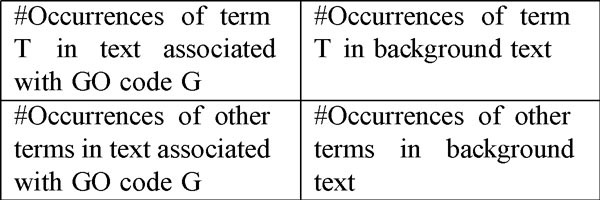
**Contingency table for the χ^2 ^test. **A high score in the test indicates that it is unlikely that the term *T *is uncorrelated with the GO code *G*.

Learning an Informative Term model involves identifying terms that are characteristic of a given GO code. To do this we separate our training set into two: a set of articles and abstracts associated with the GO code (the "support" set), and the remaining set of articles and abstracts (the "background"). Then we determine occurrence counts for each unigram, bigram and trigram in our vocabulary in the support text and in the background, and perform a χ^2 ^test on the table containing the counts, as illustrated in Figure 3. This test makes the null hypothesis that the distributions of a term in the two classes (the support and the background) are identical, and returns a score that is proportional to the strength of the alternative hypothesis. Using the returned score, we rank the terms in our vocabulary and pick those whose scores are above a threshold parameter *I *as the Informative Terms for the GO code of interest. After parameter tuning experiments on the training set, we set *I *to 200 and hold it constant for our experiments. As an example of the output of this process, for the GO code GO:0015370, *sodium symporter activity*, some of the unigram Informative Terms returned by this process are *pantothenate*, *biotin*, *transporter*, *lipoate*, *smvt*, *uptake *and *sodium-dependent*. We observe that words such as *symporter *are not significantly indicative of the presence of this GO code, though they are part of the text of the code itself. This may be because the activity in question is generally described indirectly in the text (as we might expect), by means of other words, rather than the words used in the text of the GO code.

While learning the Informative Term model for a GO code, we are able to take advantage of the hierarchical nature of the Gene Ontology in the following way. We use documents that support a GO code as support for its ancestors in the ontology as well. For example, *plasma membrane *is a direct ancestor of *integral to plasma membrane*, so, documents that are associated with *integral to plasma membrane *are used when collecting Informative Terms for *plasma membrane*. However, we decrease the weight of documents supporting each descendant GO code proportional to its depth relative to the GO code under consideration as follows. Let *P *be the GO code under consideration and let *C *be a descendant of *P*. Since each ontology is a directed acyclic graph, there may be multiple paths from *P *to *C*. Let *d*_*P*_(*C*) be the average length of a path from *P *to *C*. Then the weight *w*_*P*_(*C*) of any document supporting *C *when calculating Informative Terms for *P *is given by . Thus, documents supporting *integral to plasma membrane *would count for only half as much when calculating evidence for *plasma membrane*, since *d*_*P*_(*C*) = 1. The weight *w*_*P*_(*C*) is factored into our calculations during the χ^2 ^test. To reliably calculate statistics for the χ^2 ^test for a GO code, we need a large number of documents. In our system, we set the number of documents required at 10. For many GO codes, however, even after collecting "weakly labeled" data, we are unable to accumulate 10 documents. For such GO codes, we rely on a simple Regular Expression model. Each regular expression is built from a given GO code name and its aliases.

When given a novel document and protein, we use the Informative Term model to calculate a score for each GO code. The score is the sum of the χ^2 ^scores of all the Informative Terms that occurred in those paragraphs in the document where the protein name also occurred. A GO code is predicted to be relevant to the document if the score for that code was above a threshold parameter *S*, and further, at least *k *Informative Terms were matched. After some parameter tuning experiments on our training set, we set *S *to 4000 and *k *to 3 and held them constant for our experiments. For GO codes without Informative Term models, we use the Regular Expression model described above. A term is predicted to occur if its regular expression matches some piece of text in a paragraph where the protein name also occurs.

### Linking proteins and GO terms

Given passages of text that apparently contain references to the query protein and to Gene Ontology terms, we use a statistical model to decide which of these GO codes (if any) should be returned as annotations for the protein.

The Informative Term models do not take into account the actual words in the document beyond the Informative Terms. However, there may be words that are common to many GO codes, or that are generally indicative of text supporting the assignment of some GO code to some protein. For instance, words describing localization experiments might be characteristic of text that supports GO codes from the Component Ontology. In order to capture this evidence, we learn two Naïve Bayes classifiers [11] for each ontology. Given an attribute-value representation of some piece of text containing a match to the query protein and a GO code, these classifiers return the probability that the piece of text supports the annotation of the protein with the GO code. Therefore, they can be used to filter, or re-rank, the outputs of the Informative Term and Regular Expression models.

To learn such Naïve Bayes classifiers, we first extract features from each paragraph of the documents supporting the predictions made by the Informative Term models and the Regular Expression model on our training set. These features consist primarily of unigrams occurring in the text. We also extract several other features which capture the nature of the protein-GO code interaction, which are as follows:

1. the number of matches for the protein name,

2. the number of matches for the GO code name (or its Informative Terms),

3. the length in words of the passage starting from the first protein or Informative Term match and ending with the last such match,

4. the smallest distance in words between an occurrence of the protein and an occurrence of the GO code (or its Informative Terms),

5. the average distance in words between occurrences of the protein and the GO code (or its Informative Terms), and

6. the score of the matched GO code, if the Informative Terms model was used for this prediction.

To reduce overfitting problems, we restrict the set of unigrams used to the 200 that are most correlated with the class variable, as measured by mutual information. Given a class (supporting/not supporting), a paragraph is described as a multinomial over the vocabulary features and a product of Gaussian distributions over the numerical features:



where *D *is the paragraph, *α*_*i *_represents the conditional probability, given the class, of the *i*^*th*^word from the set of words used in *D *(Pr(*w*_*i*_|*class*)), *n*_*i *_represents the number of times *w*_*i *_occurred, and *μ*_*j *_and *σ*_*j *_represent the Gaussian parameters for the *j*^*th *^numeric feature in the given class. The parameters *α*_*i*_, *μ*_*j *_and *σ*_*j *_are then learned from the training data using Maximum Likelihood estimation. We learn Naïve Bayes models for each ontology for GO code predictions made by the Informative Term models, and separate Naïve Bayes models for each ontology for predictions made by the Regular Expression model.

### Identifying evidence text

After the GO code predictions are made by either the Informative Term model or the Regular Expression model, the corresponding Naïve Bayes model is used to score the likelihood of each paragraph of the text supporting some protein-GO code association. The maximum score over all paragraphs is used as a confidence measure for a protein-GO code association, and used to re-rank the predictions of the Informative Term and Regular Expression models. The most highly ranked GO code predictions for that protein and document are then returned by the system.

Our system focuses on predicting a GO code based on the full document given to it, rather than locating a contiguous piece of evidence text for the code. Indeed, we feel that it is a strength of our approach that we can aggregate evidence from different regions of a large document in order to make a prediction of a GO code. However, for the purposes of Task 2.1 and 2.2, we were required to identify a single piece of text that provides the best support for a predicted protein-GO code annotation. To do this, we use the following algorithm. We always return a single paragraph as evidence text. If the predicted GO code has an associated Informative Term model, we use that model to score all the paragraphs in the document where the given protein name occurred. The highest scoring paragraph is then returned. If the GO code is predicted by the Regular Expression model (or did not have an Informative Term model), we use the Naïve Bayes models described in the previous section to rank the paragraphs associated with the predictions, and we return the highest scoring one. Note that, since the Informative Term models are specific to individual GO codes, while the Naïve Bayes models are not, we expect this algorithm to return better evidence text than if we were to always use the Naïve Bayes models to select the evidence text.

## Experiments and Discussion

In this section, we present experiments evaluating our system on the initially provided training data as well as an evaluation on the test set done by the BioCreative organizers.

### Evaluation of system components

We first present experiments to evaluate the contribution of components of our system: the Informative Term models, the Regular Expression models and the Naïve Bayes models. For evaluation purposes, we separate the set of documents given to us into a training and a test set. Since we have documents from the *Journal of Biological Chemistry *(*JBC*) and *Nature*, there is a natural partition of the set of documents. During development, we use the *JBC *documents as a training set for our Informative Term and Naïve Bayes models. We use the *Nature *documents as a test set. Thus, we learn Informative Term models from the known GO code annotations associated with the *JBC *documents. Then, we use these models and the Regular Expression model to make predictions of GO codes on the *JBC *documents. We extract features from these predictions and learn Naïve Bayes models using these extracted features. For evaluation, we use the Informative Term models and Regular Expression model to make predictions on the documents from the *Nature *journals. We then use our learned Naïve Bayes models to rank and filter predicted GO annotations for given proteins and documents.

To evaluate the predictive accuracy of our models, we measure the precision, recall and false positive rates of our predictions. Precision is the fraction of predicted GO code annotations that are correct. Recall (also referred to as the "true positive rate") is defined as the fraction of correct GO code annotations that are predicted by the system. The false positive rate is defined as the fraction of negative examples that were incorrectly predicted as positive.

First, we evaluate the performance of the Informative Term and Regular Expression models. For this experiment, we do not use the Naïve Bayes models. We use the Informative Term models and the Regular Expression model to predict GO codes from the *Nature *articles, and measure precision and recall. These results are shown in Table 1. For the "Combined" experiment, we combine the two models in the following way: we use the Informative Term model for every GO code that has such a model, and use the Regular Expression model for any GO code without an Informative Term model. Note that this implies that the recall of the "Combined" system will not be the sum of the recall of the two systems used independently. From the results, we observe that without the Naïve Bayes models, the system is biased towards recall at the expense of precision. However, we expect to improve our precision by re-ranking these initial predictions using the Naïve Bayes models and thresholding the associated confidence. Note that the recall shown for the "Combined" system in Table 1 is the maximum recall achievable by the system.

**Figure 4:**
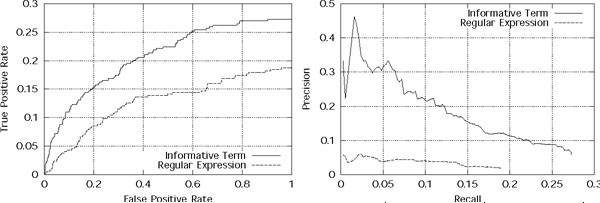
**ROC and precision-recall graphs for predictions made by the system. **ROC and precision-recall graphs for GO code predictions made by the system aggregated over the three ontologies. The solid line represents predictions made by the Informative Term/Naïve Bayes path in Figure 1. The dashed line represents predictions made by the Regular Expression/Naïve Bayes path in Figure 1.

To measure the value of re-ranking predictions using the Naïve Bayes models, we construct precision-recall (PR) and receiver operating characteristic (ROC) graphs. ROC graphs measure the change of the true-positive rate (recall) of the model against its false-positive rate as a threshold is moved across a measure of confidence in the model's predictions. PR graphs measure the change in precision at different levels of recall. We assess the confidence of a GO code annotation for a protein given a document as the maximum of the probabilities defined by Equation 1 over the paragraphs of the document. In Figure 4, we show PR and ROC graphs for these models for predictions aggregated over the Component, Function and Process ontologies. We show separate graphs for the cases when the initial predictions were made by the Informative Term models and when the initial predictions were made by the Regular Expression model. In the ROC graphs, the true positive rate (recall) values are scaled to reflect the fact that the maximum recall achievable by the Naïve Bayes system is limited by the predictions of the GO code models used in the first phase.

From Figure 4, we observe that the Naïve Bayes models are quite effective at re-ranking the initially hypothesized annotations, especially when the initial predictions are made by the Informative Term models. Annotations with higher Naïve Bayes scores are more likely to be correct. Thus, these models are useful in discriminating between passages of text that relate proteins to GO codes from those that do not. We also observe that when the initial predictions are made by the Regular Expression model, the Naïve Bayes models do not achieve high precision, even at high confidence thresholds. This indicates that (i) there may not be much regularity to be captured in passages supporting these predictions, and/or (ii) our training assumption, that each paragraph in the supporting text mentioning the protein and the GO code was actually relating the protein to the GO code, was severely violated. We note that, if we had sufficient training data for each GO term, we would not need to use the Regular Expression model at all. Thus, in the limit of sufficient training data, the behavior of the system is predicted by the graphs for the Informative Term models.

### Utility of weakly labeled data sources

To learn models for GO codes, we collect "weakly labeled" data from public databases for various other organisms, such as SGD, FlyBase, WormBase and TAIR. This data is in the form of PubMed abstracts of articles referred to by GO annotations mentioned in these databases. It is "weakly labeled" because an abstract may not mention the association between a GO code and a protein of interest, or even mention the protein. An interesting question to ask is whether this data benefits our system by making it more accurate. In this section, we describe an experiment to answer this question.

In this experiment, we evaluate a version of our system which is trained on the provided *JBC *journal articles only. Thus, Informative Term models are built only for those GO codes that have sufficient coverage in the *JBC *journal articles. For our original system, the coverage threshold was 10 documents, i.e. a GO code had to be annotated with at least 10 documents (full text or abstract) in order for us to build an Informative Term model for it. For this experiment, we used a coverage threshold of 3 documents. Even with a lower threshold, we could not build Informative Term models for over 200 GO codes for which we had models in our original system.

**Figure 5:**
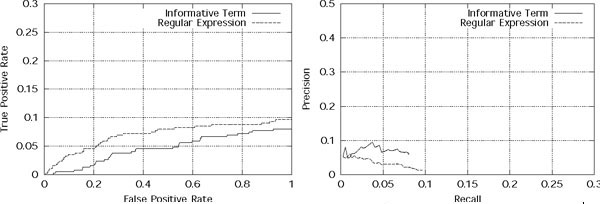
**ROC and precision-recall graphs for predictions made without weakly-labeled data. **ROC and precision-recall graphs for GO code predictions made by the system aggregated over the three ontologies, when Informative Term models were learned without using any weakly labeled data. The solid line represents predictions made by the Informative Term/Naïve Bayes path in Figure 1. The dashed line represents predictions made by the Regular Expression/Naïve Bayes path in Figure 1.

The PR and ROC graphs for this system, tested on the held-out *Nature *articles, are shown in Figure 5. Comparing these results to Figure 4, we observe that the performance of the system has degraded significantly over the original system. In fact, the Informative Term models now have lower recall than the Regular Expression model! Not only does the maximum recall drop to 18.1% as compared to 45.7% previously, the system's end-point precision also decreases to 1.97% from 2.9%. The lower performance can be attributed to two causes: first, fewer GO codes have Informative Term models, so that the lower-precision Regular Expression model is used more often. Second, even for the GO codes that have Informative Term models, the informative terms are likely to be of lower quality because the document coverage threshold is lower. Because of these factors, the predictions used to learn the Naïve Bayes models are also of lower quality. This reduces the Naïve Bayes models' ability to effectively re-rank the test set predictions.

Thus, from this experiment, we conclude that using the weakly labeled data as part of our training set is very important in increasing both the precision and the recall of our system.

### Evaluation on test data

**Figure 6:**
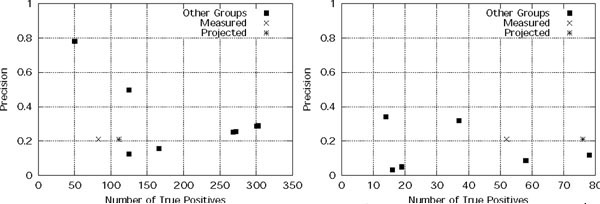
**Scatterplots of predictions for various systems for Task 2.1 (left) and Task 2.2 (right). **Scatterplots for GO code predictions made by the various systems on the test set for Task 2.1 (left) and Task 2.2 (right). The graphs plot system precision against the number of true positive predictions (we do not plot recall because the total number of positive instances is unknown).

In this section, we discuss the performance of our system relative to the other submitted systems on the test set data, as evaluated by the BioCreative organizers. We should note that because of technical difficulties during evaluation, our complete submission for this task was not evaluated. It is possible that our recall may have improved if this evaluation were done. Accordingly, we have plotted a "Projected Recall" point in Figure 6 which indicates the estimated recall of our system had our full submission been evaluated, assuming that our precision stays at the reported level.

The results for the various systems are shown in Figure 6 for Tasks 2.1 and 2.2. We plot the precision of each system against the number of true positive predictions made. Unlike the training set, the total number of correct GO code assignments is unknown for the test set. Therefore, we do not use recall; however, the number of true positive predictions is proportional to recall. If multiple runs were evaluated for a group, we plot the best result from that group. It is clear that no system provides exceptional results for the overall task, and there is much room for improvement. We observe that for Task 2.2, our system is able to achieve a reasonable compromise between precision and recall – it has the third best precision as well as the third best recall. Further, our (estimated) projected recall is the second highest among all the groups. For Task 2.1, the test set results for our system are not as good. However, as we have already noted, this is likely because our system concentrates on modeling the full text of the article rather than any specific passage, and we were asked to report a specific passage for this task.

## Conclusion

We have built a system that uses learned statistical models to automatically annotate proteins with codes from the Gene Ontology based on articles from the scientific literature. Our experimental evaluation of the system indicates that it has predictive value. In particular, our experiments show that the use of weakly labeled data sources can significantly improve the precision-recall characteristics of systems for this annotation task. However, there is still much room for improvement. In future work, we plan to investigate several key issues including (i) learning edit-distance based models for recognizing additional instances of protein names, (ii) using models with linguistically richer representations for the step of filtering and ranking candidate annotations, and (iii) using a *multiple-instance *based approach [12] when learning models for filtering and ranking. The application of a multiple-instance approach is motivated by the fact that, even in the training data, the passages of text that support a given annotation are not marked.

## Figures and Tables

**Table 1:** Precision and recall results for the system without the Naïve Bayes models.

Experiment	Precision(%)	Recall(%)
Regular Expression	2.1	21.0
Informative Terms	6.1	27.7
Combined	2.9	45.7
